# Neurocysticercosis and cerebral hemodynamic changes: can transcranial Doppler be the key?

**DOI:** 10.1055/s-0045-1804918

**Published:** 2025-03-25

**Authors:** Prateek Kumar Panda, Indar Kumar Sharawat

**Affiliations:** 1 All India Institute of Medical Sciences, Department of Pediatrics, Division of Pediatric Neurology, Rishikesh Uttarakhand, India.

Dear Editor,

We read with interest the article titled “Transcranial Doppler Ultrasonography to Evaluate Cerebral Hemodynamic Changes in Neurocysticercosis,” by Sanchez-Boluarte et al. 1 The authors reported that cerebral hemodynamic changes suggestive of vasculitis are frequent in neurocysticercosis (NCC) patients and can be evaluated using transcranial Doppler (TCD). However, we would like to raise a few concerns.

The study describes participants' age, gender distribution, education level, type of NCC (parenchymal/subarachnoid), use of steroids, and illness duration in relation to the presence of vasculitis. However, key variables such as the stage of NCC, lobar location, number of lesions, and seizure occurrence were not reported or correlated with vasculitis. Including these factors would provide a more comprehensive analysis. Furthermore, the authors noted differences in sociodemographic variables between parenchymal and subarachnoid NCC groups but did not perform multivariate logistic regression to determine which variables were independently associated with vasculitis. 2 Adding these analyses would enhance the article's clinical relevance.

Additionally, the study included only patients with a positive enzyme-linked immunoelectrotransfer blot (EITB) test. However, according to the 2017 guidelines of the Infectious Diseases Society of America (IDSA), the sensitivity of serum EITB is nearly 100% in patients with multiple parenchymal, ventricular, or subarachnoid NCC but is lower in those with a single parenchymal lesion or calcifications. 3 As a result, the authors may have excluded a significant number of patients with single lesions. It would be helpful to know how many suspected NCC cases were excluded due to negative serology to assess the potential for selection bias.

Previous studies have used magnetic resonance imaging (MRI), magnetic resonance angiography (MRA), and computed tomography (CT) angiography to diagnose cerebral vasculitis in NCC patients, often finding cerebral infarcts and hydrocephalus in these cases. 4 The authors did not report any MRI or MRA findings, which would have allowed for comparison with the sensitivity and specificity of TCD in detecting cerebral vasculitis. While TCD is a useful, non-invasive tool to monitor vasculitis, it is not the most sensitive method for detection. By not incorporating MRI or MRA data, the study may have underestimated the prevalence of vasculitis.

Finally, the authors did not account for other variables associated with vasculitis, such as antinuclear antibody (ANA) positivity, age, atherosclerosis, or the use of drugs like cocaine. 5 Addressing these factors would strengthen the conclusion that vasculitis was solely due to NCC, especially given the lack of a control group in the study.
